# Stress, burnout and doctors' attitudes to work are determined by personality and learning style: A twelve year longitudinal study of UK medical graduates

**DOI:** 10.1186/1741-7015-2-29

**Published:** 2004-08-18

**Authors:** IC McManus, A Keeling, E Paice

**Affiliations:** 1 Department of Psychology, University College London, Gower Street, London WC1E 6BT, United Kingdom; 2 London Department of Postgraduate Medical and Dental Education, 22 Guilford Street, London WC1N 1DZ, United Kingdom

## Abstract

**Background:**

The study investigated the extent to which approaches to work, workplace climate, stress, burnout and satisfaction with medicine as a career in doctors aged about thirty are predicted by measures of learning style and personality measured five to twelve years earlier when the doctors were applicants to medical school or were medical students.

**Methods:**

Prospective study of a large cohort of doctors. The participants were first studied when they applied to any of five UK medical schools in 1990. Postal questionnaires were sent to all doctors with a traceable address on the current or a previous *Medical Register*. The current questionnaire included measures of Approaches to Work, Workplace Climate, stress (General Health Questionnaire), burnout (Maslach Burnout Inventory), and satisfaction with medicine as a career and personality (Big Five). Previous questionnaires had included measures of learning style (Study Process Questionnaire) and personality.

**Results:**

Doctors' approaches to work were predicted by study habits and learning styles, both at application to medical school and in the final year. How doctors perceive their workplace climate and workload is predicted both by approaches to work and by measures of stress, burnout and satisfaction with medicine. These characteristics are partially predicted by trait measures of personality taken five years earlier. Stress, burnout and satisfaction also correlate with trait measures of personality taken five years earlier.

**Conclusions:**

Differences in approach to work and perceived workplace climate seem mainly to reflect stable, long-term individual differences in doctors themselves, reflected in measures of personality and learning style.

## Background

Sir William Osler (1849–1919), one of the most distinguished physicians of the nineteenth and early twentieth century, recognised that only some doctors are happy in their professional lives:

"To each one of you the practice of medicine will be very much as you make it – to one a worry, a care, a perpetual annoyance; to another, a daily joy and a life of as much happiness and usefulness as can well fall to the lot of man."[1]

The modern medical workplace is a complex environment, and doctors respond differently to it, some finding it stimulating and exciting, whereas others become stressed and burned out from the heavy workload. The medical workplace also provides an environment where new skills are continually being learned, both as a result of medical knowledge evolving and because a doctor's work changes, in part due to career development and progression through different jobs.

In an important study, Delva *et al *[2] used earlier research [3,4] to develop two separate instruments for studying how doctors work, the Approach to Work Questionnaire (AWQ) and the Workplace Climate Questionnaire (WCQ). In Canadian physicians [2,5] the AWQ showed three separate factors, which were called *Surface-Rational*, *Surface-Disorganised*, and *Deep *(see table 1). These approaches related to different methods and motivations for continuing medical education. Those with a deep approach preferred independent and problem-based learning and motivation was internal. Surface-rational and surface-disorganised approaches were primarily driven by external motivation, with the preferred mode of continuing education learning being independent for the surface-rational, and in consultations for the surface-disorganised.

The WCQ showed three dimensions, called *Choice-Independence, Supportive-Receptive*, and *Workload *(see table 1), which correlated with the AWQ. Doctors reporting Choice-Independence and Supportive-Receptive work environments had a Deeper approach, whereas those describing an environment dominated by Workload tended to be more Surface-Disorganised.

Some doctors are unhappy with their work, which can manifest as stress (usually assessed by the General Health Questionnaire) or burnout, which has three separate components of emotional exhaustion, depersonalisation and reduced personal accomplishment (see table 2). Greater stress and burnout in doctors are related to the personality trait of neuroticism or 'negative affectivity' [6].

The AWQ and WCQ provide a snapshot of a doctor's learning environment and approach to work at one particular time, as also do measures of stress and burnout. A key question, as Deary *et al *recognised [6] when considering stress, is the extent to which different approaches to work and the climate of the workplace are consequences *of the workplace *or *of the doctor*. At first sight it might seem that the workplace itself has to be the primary force driving both workplace learning and workplace climate. However, it is also possible that approaches to learning and work mainly depend upon pre-existing differences among doctors, differences that may already have manifested earlier in the doctors' careers. The AWQ bears a strong formal similarity to the surface, deep and strategic study habits and learning styles identified by the Study Process Questionnaire (SPQ), which assesses the motivations and approaches used by students in higher education (see table 3). The similarity is not accidental since the AWQ was developed by adapting items from Entwistle and Ramsden's Approaches to Study Inventory [7], which has a similar factor structure to that of the Study Process Questionnaire [8]. It is therefore expected that there may be significant continuities across approaches to study and approaches to work.

In this paper we describe a large cohort of UK doctors, typically aged 29 or 30 at the time of the study, who have been qualified for five or six years, who are practising as SHOs or SpRs in hospital or are in general practice, and who previously had been studied when aged 17 or 18 at application to medical school in autumn 1990 [9], in their final year at medical school [10] and as PRHOs [11]. The main interest here will be in the extent to which a doctor's present approaches to work and their workplace climate, as well as their stress and burnout, relate to earlier measures of study habits and personality at application to medical school and subsequently.

## Method

### Participants

**Figure 1:**
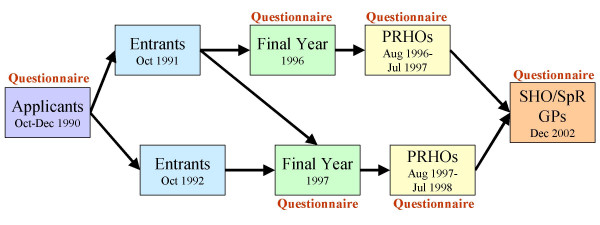
Diagram showing the timing of the various stages of the survey. Note that not all applicants entered in October 1991 (despite in most cases having applied to do so), and subsequent stages of the study therefore took place at different times. Some entrants also took a year longer because of taking an intercalated degree (represented by the diagonal arrow from entrants in Oct 1991 to Final year in 1997), and a few other students delayed for other reasons. All doctors were surveyed at the end of their PRHO year, whenever that had occurred. The cohort was finally brought back into step as a single cohort with the 2002 follow-up when all doctors were studied at the same time, irrespective of the time at which they qualified. Questionnaires were given out at all the boxes shown (with the exception of a questionnaire to Entrants, who are shown merely to make the flow clearer). It should also be noted that there was a follow-up of a subset of the students in their third academic year, which is not shown here because the data are not discussed in this study.

In the autumn of 1990 a questionnaire was sent to all individuals with European Community postal addresses who had applied to any of the five UK medical schools taking part in the study [9]; they represented about 70% of all applicants and acceptances for medical school in that year. The response rate was 93%. Students who were accepted for entry in 1991, 1992 or 1993 were followed up in their final year at medical school (1995–1998), when the response rate was 56%, and at the end of their PRHO year (1996–1999), when the response rate was 58%. In 2002 a tracing exercise searched the *Medical Register *and *Medical Directory *from 1995 to 2002 to find the addresses of as many doctors as possible who were in the original survey, and who were known not to have died, left medical school during basic medical sciences, or otherwise to be no longer in the survey. For study design see figure 1. 

### Questionnaire

Questionnaires were sent to all individuals with current or recent GMC addresses. The questionnaire consisted of a single folded A3 sheet of paper (4 A4 sides). Included in the present questionnaire (described in the results as '2002') were the 12-item General Health Questionnaire (GHQ) [12]; an abbreviated version of the Maslach Burnout Inventory (aMBI), which has three sub-scales, Emotional Exhaustion, Depersonalisation, and Personal Accomplishment [13,14]; a three-item scale modelled on the aMBI, which assesses Happiness with a Medical Career [15]; an abbreviated version of the Study Process Questionnaire, which has three sub-scales of Surface, Strategic and Deep learning [16,17]; an abbreviated questionnaire assessing the 'Big Five' personality dimensions of Neuroticism, Extraversion, Openness to Experience, Agreeableness and Conscientiousness [15,18]; and abbreviated versions of the Approach to Work Questionnaire (aAWQ) and the Workplace Climate Questionnaire (aWCQ) [19], each of which has three sub-scales, and for which a detailed description is provided in the **Supplementary Information** (see Additional file: 1) [2]. The GHQ, aMBI and personality questionnaire had also been administered previously in the PRHO survey, and the SPQ had been administered in the Applicant and Final year surveys.

### Procedure

Questionnaires, along with a postage-paid return envelope, were posted at the beginning of December 2002. Two reminders were sent to non-respondents. Although the official closing date was 25^th ^March 2003, a few questionnaires were returned up until the end of August 2003.

Statistical analysis used SPSS version 10.5, and structural equation modelling used LISREL 8.52.

## Results

The tracing exercise looked for 2,912 individuals thought to have completed basic medical sciences and entered a clinical course. Eighty-nine had never been on the UK Medical Register, and either had failed finals, had never registered, or had emigrated. Of 2,823 individuals who were traced, 2,754 doctors were on the 2002 Register, 7 returned to the Register during 2002, and 64 were on an earlier Register. Of 2,823 questionnaires sent, 176 were returned by the Post Office as undeliverable, 10 doctors were travelling and hence uncontactable, and2 had died. Of the remaining 2,635 doctors, 1,668 returned questionnaires, giving a response rate of 63.3%. There was no evidence of response bias (see **Supplementary Information** see Additional file: 1).

### Respondents

The mean age of respondents on 1^st ^December 2002 was 30.4 years (SD 1.86, range 28.3 – 49.2). There was substantial variation in the scores on the aAWQ and the aWCQ, and the factor structures of the aAWQ and aWCQ were similar to those reported elsewhere [19] (see **Supplementary Information** see Additional file: 1). There was also substantial variation on the measures of stress, burnout and satisfaction with medicine as a career, with 21.3% of doctors (345/1617) reporting GHQ scores of 4 or more, the conventional level of 'caseness'.

Approaches to work and learning were correlated with climate in the workplace, and as in the Delva *et al *study, the highest correlations were for a surface-disorganised approach correlating with high workload, and a deep approach correlating with a supportive-receptive environment and with choice-independence (table 4).

### Approaches to work

Table 4 shows correlation of the stress measures with approaches to work and study habits. The largest correlations were of a surface-rational approach with a strategic learning style, and a deep approach to work with a deep learning style. In each case the correlations were not only highly significant when study habits were measured in the final year at medical school, six or seven years earlier, but were also very significantly correlated with study habits measured at selection, twelve years earlier. Correlations of approaches to work and stress, burnout and satisfaction with medicine were generally small, and generally were only with measures taken in 2002, and not with measures taken as a PRHO, five or six years earlier. The sole exception was that a surface-disorganised approach correlated with high stress as measured by the GHQ, both in 2002 and with stress when the doctors were PRHOs.

### Workplace climate

Table 5 shows correlations between the workplace climate and study habits, stress, burnout and satisfaction with medicine. In contrast to the associations with approaches to work, the workplace climate showed only small correlations with study habits, but showed strong correlations with stress, burnout and satisfaction with medicine. In particular, high stress in the PRHO year showed very significant correlations with measures in 2002 of a perceived high workload, a less supportive-receptive environment, and less choice-independence. In addition, emotional exhaustion both in 2002 and in the PRHO year were related to a high perceived workload in 2002.

### Personality

Table 6 shows the correlations of approaches to work and workplace climate with the 'Big Five' measures of personality, measured both in 2002 and also measured five to six years previously when the doctors were PRHOs. The surface-disordered approach to work is associated with high neuroticism and low conscientiousness, the PRHO correlations also being highly significant in each case. Neuroticism, both in 2002 and as a PRHO, is also associated with a perceived high workload (although in contrast to its prediction of a surface-disordered approach, conscientiousness is not a significant correlate of workload). The deep approach to work and learning is associated with being extravert and with greater openness to experience, and again the measures taken six years earlier are predictive. Finally a supportive-receptive work climate is associated with greater reported agreeableness, both in 2002 and six years earlier as a PRHO. There were no substantial correlations between personality and the surface-rational approach to work or choice-independence in work climate.

### Multiple regressions

Tables 4 to 6 show a large number of correlations, which are not always straightforward to interpret, both because they are numerous and because many variables are themselves inter-correlated. Multiple regression was used to clarify the relationships (for technical details see **Supplementary Information** see Additional file: 1). Each individual measure of the aAWQ and aWCQ was regressed on the measures of study habits at application (n = 3) and in the final year (n = 3), of stress and burnout during the PRHO year (n = 4) and in 2002 (n = 4), and of personality in the PRHO year (n = 5) and in 2002 (n = 5). Alpha for entry was set at p < 0.0001 in view of the large sample size and the number of independent variables. The variables that were significant are shown in tables 4,5 and 6 and 3 in italics. Of particular interest are variables that show not only show significant contemporaneous correlations but also significant correlations when measured five or more years previously.

A surface-disorganised approach to work is predicted by surface learning in medical school and by higher neuroticism scores and lower conscientiousness (see tables 4 and 6). The surface-rational approach to work is predicted by strategic learning in medical school, and by less openness to experience and higher conscientiousness. The deep approach to work is predicted by a deep approach to learning at medical school, by greater extraversion, by greater openness to experience, and by lower emotional exhaustion.

A workplace climate dominated by a high workload is predicted by higher stress and emotional exhaustion measures five years earlier, and by lower openness to experience (see tables 5 and 6). A supportive-receptive workplace is predicted by lower stress and depersonalisation, and a higher sense of personal accomplishment when measured previously, and by a more agreeable personality. Choice-independence in the work environment is predicted only by lower previous measures of stress.

### Stress, burnout and satisfaction with medicine

Although in the previous analyses, stress and burnout have been used as predictors of approaches to work and workplace climate, they are also important outcome measures in their own right. Table 7 shows the correlations of the five 'stress-related measures' (GHQ, the three burnout measures and satisfaction) with measures of learning style and personality, in each case measured on two separate occasions. Personality correlates with each of the measures, as do study habits. Because of the complex inter-correlations between the dependent variables, multiple regression was used, as before, to find the most important relationships (for technical details see **Supplementary Information** see Additional file: 1). Doctors who are most stressed showed higher levels of neuroticism, both currently and previously, and those reporting most emotional exhaustion also had higher neuroticism levels, as well as being more introvert. High levels of depersonalisation related to lower levels of agreeableness. A greater sense of personal accomplishment related to previous deep approaches to study and learning, as well as to being more extravert. Overall satisfaction with medicine as a career related to lower levels of neuroticism.

### Path analysis

**Figure 2:**
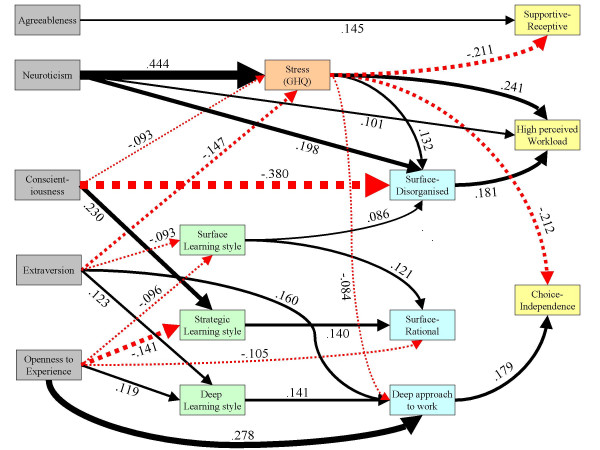
Path diagram showing the relationships among the measures of personality, learning style, stress, approaches to work, and workplace climate. The width of arrows is proportional to the strength of an effect, which is shown alongside each line as a path (beta) coefficient. Negative effects are shown as red, dashed lines. For details of the statistical method and a fuller model incorporating all links, see **Supplementary Information**.

The complex relationships described by the various correlations are best analysed and described by means of path analysis or causal modelling [20], which analyses the entire set of correlations between variables, using plausible assumptions about causality and removing non-significant paths. The path diagram, which was analysed using LISREL 8.52 [21], is shown in figure 2. Measures to the left can causally influence measures to their right. Based on the time-lagged correlations reported previously, we assumed that stress causes different approaches to work, and we also assumed that approaches to work cause differences in workplace climate rather than vice-versa. (Nevertheless, we acknowledge that the causation may well be reciprocal, as suggested by the originators of the scale [2,19]; further longitudinal data will be required to test that hypothesis). Study habits are temporally and causally prior to stress, approaches to work and workplace climate. Personality, being a trait, was prior to all other measures. For technical details see the **Supplementary Information** (see Additional file: 1). Although several of our variables are measured at different time points, we have chosen not to present a model in which each variable has been included on each occasion that it is measured, as the resulting diagram becomes unmanageably complex.

Although the path diagram in figure 2 is complex at first sight, the paths are readily interpretable. The diagram divides into two broad sections, with the measures of learning style and approach to work at the bottom, and stress at the top. Here we have simplified the model by omitting the closely correlated measures of burnout, and only including paths with t-values greater than 3.6. Estimates of all the paths are available in the **Supplementary Information** (see Additional file: 1).

Stress in our model is caused by personality differences, being greatest in those having high neuroticism scores, low extraversion scores, and low conscientiousness scores. It is unrelated to learning style.

Learning styles at medical school relate to different personality measures, in particular showing no relationship to neuroticism. Deep learning is highest in extraverts who are open to experience, whereas strategic learning is highest in highly conscientious individuals with low openness to experience. Surface learning style is higher in introverts who are low in openness to experience. These findings are similar to those of others [22].

Approaches to work are mainly but not entirely driven by learning styles. A deep approach to work occurs in extraverts who are open to experience and have a deep learning style. The surface-rational and surface-disorganised approaches to work are both greater in those with a surface learning style. However, a surface-disorganised approach occurs in individuals with higher neuroticism scores, in those with lower conscientiousness scores, and in those who have been stressed, whereas the surface-rational approach to work occurs in strategic learners and in those who are low in openness to experience.

Workplace climate has a range of influences. High perceived workload occurs in those with a surface-disorganised approach to work, who have been stressed and are more neurotic. In contrast, choice-independence and a supportive-receptive environment both occur in individuals who have not previously been stressed, the choice-independence approach occurring in those with a deep approach to work, whereas the supportive-receptive approach occurs in those who have higher scores on the personality trait of agreeableness.

## Discussion

Many doctors at the age of 30 are unhappy in their jobs, and a fifth of our sample reached the conventional GHQ criterion of psychiatric 'caseness'. In contrast, many doctors reported high levels of personal accomplishment, choice and independence in their work environment, satisfaction with medicine as a career, and intellectual and emotional satisfaction from their work. That is not new; Sir William Osler in 1905 contrasted doctors "whose stability of character and devotion to duty make one proud of our profession" with those who find it difficult to keep "the flame alive, smothered as it is apt to be by the dust and ashes of the daily routine" [1].

In 2001, Richard Smith asked "Why are doctors so unhappy?" and concluded that "The most obvious cause of doctors' unhappiness is that they feel overworked and undersupported" [23]. Certainly many doctors in our study report a high workload and a work climate that is neither supportive nor receptive, and those doctors also report more stress, burnout and dissatisfaction with medicine as a career. It is tempting therefore to conclude, as did an article in a special edition of *BMJ Careers *devoted to "Doctors' Wellbeing", that excessive workload and absence of support are directly caused by poor working conditions: "the way in which the NHS is run generates stress for members of the workforce every day" [24]. However, such an interpretation is not straightforward in general [25]. It is particularly difficult for the doctors in our study because the study is longitudinal, and workload and lack of support correlate *with stress and burnout reported five or six years earlier*, when the doctors were PRHOs and carrying out entirely different jobs. High perceived workload and poor support are therefore determined as much by doctors themselves as by specific working conditions. That view was expressed in another article in the special edition of *BMJ Careers*: "A critical element contributing to the stress that many conscientious doctors experience is internal..." [26]. A similar conclusion was reached in a previous study of ours when these doctors were PRHOs, and multi-level modelling showed that stress is not a characteristic of jobs but of doctors, different doctors working in the same job being no more similar in their stress and burnout than different doctors in different jobs [11].

If differences in reported workload are partly explained by differences among doctors, what in turn explains those differences? Doctors reporting a high workload also have what Delva *et al *[2] describe as a *surface-disorganised *approach to work, which in turn is correlated with being a surface learner at application to medical school, a dozen years previously. Surface-disorganised doctors are also high on the personality trait of neuroticism and low on the trait of conscientiousness; and again those correlations are with measures taken six years earlier when the doctors were PRHOs. Doctors reporting a work climate low in support were lower on the personality scale of agreeableness in the measures collected when they were PRHOs.

Some doctors may be stressed and burned out, but what predicts those others who are happy in their work? Doctors reporting high satisfaction with medicine as a career have a deep approach to work, and that approach is more common in those who also had a deep learning style when they applied to medical school. Satisfaction with medicine also relates directly to the personality traits of greater extraversion and lower neuroticism, and the deep approach to work correlates with greater extraversion and more openness to experience. Doctors who describe their colleagues as receptive and supportive score more highly on the personality trait of agreeableness; and as in many other correlations reported here, that correlation is stable across time – those who are more agreeable at the age of 24 have a more receptive and supportive work environment when aged 30.

An overview of our findings is that approaches to work are predicted by earlier measures of study habits and learning styles, whereas perceived work climate, and its pathologies such as stress and burnout, are predicted mainly by personality. Although unfortunately our study did not measure personality during selection, the high stability of the Big Five measures across the life-span [27-29] (and across our two measures six years apart), as well as their heritable component [30], means that we have little doubt that personality at selection would also have been predictive, particularly given that a similar pattern of correlations was found in a different cohort of doctors in mid-career [15]. Other studies on very different groups of students have also found, like us, that both strategic and deep learning correlate with conscientiousness, and that deep learning also correlates with extraversion and openness to experience [22,31]. Our study has, for various reasons, not looked at academic performance in relation to study habits, learning styles and personality, although previous work of ours has found clear correlations between learning styles and examination performance [32]. In contrast we have not found any correlation of undergraduate or postgraduate academic achievement with personality [15], and although some studies have found correlations of conscientiousness with academic achievement [33], this does seem to vary according to the learning context [34,35]. Although we will be looking at this question again in more detail in a further analysis, it does seem probable that personality mostly has an indirect effect upon academic achievement via approaches to learning [31,36].

If, as William Wordsworth said, "the child is father to the man", then the seeds of subsequent job satisfaction and dissatisfaction in doctors may be visible in the personality, motivations and learning styles of medical school applicants. This argument may provide some justification for using such measures in selection, particularly given the general association of job performance and satisfaction with personality [37] and motivation [38], and learning styles with personality [22] .

However, just as genes are not destiny, so neither personality nor learning style is destiny. Nurture interacts with nature [39], the environment building upon the genes, and the genes using what is provided by the environment; the poetic complement to William Wordsworth is therefore Alexander Pope, who said, "This education forms the common mind: Just as the twig is bent, the tree's inclined." Extreme introverts can, with sufficient insight, preparation and appropriate training become effective public speakers, less conscientious individuals can learn to be more organised and efficient, and those who are more neurotic can transcend their anxieties (and indeed neuroticism may be beneficial if sublimated into a professional concern for detail in critical situations, rather than merely being undifferentiated personal anxiety). .

Formal education, particularly effective formal education [40], can also alter study habits and learning styles, which are less fixed and 'trait-like' than personality measures [17]. Intercalated degrees increase deep and strategic learning and decrease surface learning at medical school [41], making it likely that they also encourage surface-rational and deep approaches to work. Deep and strategic learning also relate to the clinical experience gained by medical students [32], making it possible that greater patient involvement during undergraduate clinical training, rather than mere reliance on textbook learning to pass exams, a characteristic of surface learners, will also reduce surface-disorganised approaches to work.

## Conclusions

Longitudinal data suggest that personality and learning style are not merely *correlates *of approaches to work, workplace climate, stress, burnout and satisfaction with a medical career, but are *causes*, events later in time being predicted by events earlier in time [35]. Doctors with greater stress and emotional exhaustion, who were less satisfied with medicine as a career, had higher neuroticism scores and were more likely to be surface-disorganised. Lower conscientiousness on the personality measure also predicted greater stress. Extraverts reported more personal accomplishment and were more satisfied with medicine. The personality measure of agreeableness predicted a more supportive-receptive work environment.

These results imply that differences in approach to work and workplace climate in our study result from differences among doctors themselves, as much as they do from differences in working conditions.

## Competing interests

None declared.

## Authors' contributions

The cohort study was designed by ICMcM. The present follow-up was designed by ICMcM and EP, who also prepared the questionnaire. AK was responsible for day-to-day running of the study, and for data entry and cleaning. ICMcM was primarily responsible for data analysis and for writing the first draft of the paper. ICMcM, EP and AK were all involved in preparing the final draft of the paper.

## Pre-publication history

The pre-publication history for this paper can be accessed here:



## Supplementary Material

Additional file 1 Click here for file

## Figures and Tables

**Table 1:** Dimensions of the AWQ and WCQ [2].

**Approaches to work questionnaire**	
*Surface disorganised:*	Feeling overwhelmed by work. For example, being unsure what is needed to complete a task, finding it difficult to organise time effectively, reading things without really understanding them.
*Surface rational:*	Preference for order, detail, and routine. For example likes to know precisely what is expected, puts of a lot of effort into memorising important facts when learning something new.
*Deep approach:*	Integrative approach that leads to personal understanding. For example, tries to relate new ideas to situations where they might apply.

**Workplace climate questionnaire**	

*Choice-independence:*	Perception of control over what one does and how one does it.
*Supportive-receptive:*	Perception that help is available in the workplace and colleagues are understanding.
*Workload:*	Perception of heavy workload and having to cope alone.

**Table 2:** The three separate components of burnout [42]. *Note*: burnout on the MBI is indicated by higher scores on the emotional exhaustion and depersonalisation scales, and lower scores on the scale of personal accomplishment.

*Emotional exhaustion:*	Reduced energy and job enthusiasm; emotional and cognitive distancing from the job.
*Depersonalisation:*	Cynicism; lack of engagement and distancing from patients; treatment of patients as inanimate, unfeeling objects.
*Personal accomplishment:*	A sense of efficacy and effectiveness; of involvement, commitment and engagement; of capacity to innovate, change and improve.

**Table 3:** Differences in motivation and process of the surface, deep and strategic approaches to learning assessed in the Study Process Questionnaire [41].

	**Motivation**	**Process**
**Surface**	Completion of the course Fear of failure	Rote learning of facts and ideas Focussing on task components in isolation Little real interest in content
**Deep**	Interest in the subject Vocational relevance Personal understanding	Relate ideas to evidence Integration of material across courses Identifying general principles
**Strategic**	Achieving high grades Competing with others To be successful	Use techniques that achieve highest grades Level of understanding Patchy and variable

**Table 4:** Pearson correlation (Significance; N) of background measures with the Approaches to Work Questionnaire. Correlations significant at p < 0.05 are in bold, and those with an absolute value of greater than 0.2 are underlined. Variables that are significant in the multiple regression (see text) are indicated in italics.

	Time of measurement	aAWQ Surface-Disorganised	aAWQ Surface-Rational	aAWQ Deep
aWCQ Workload	2002	**0.259** (p < .001; N = 1644)	0.045 (p = .071, N = 1642)	**-0.068 **(p = .006, N = 1638)
aWCQ Supportive-Receptive	2002	**-0.112 **(p < .001; N = 1636)	**0.064 **(P = .009; N = 1633)	**0.102 **(P < .001; N = 1630)
aWCQ Choice-Independence	2002	**-0.125 **(P < .001; N = 1647)	-0.001 (P = .976; N = 1645)	**0.209** (P < .001; N = 1641)
SPQ Surface Learning	Application	***0.119 ***(P < .001; N = 1591)	**0.105 **(P < .001; N = 1589)	**-0.081 **(P < .001; N = 1584)
	Final Year	**0.105 **(P = .001; N = 999)	**0.101 **(P = .001; N = 1001)	**-0.109 **(P = .001; N = 996)
SPQ Strategic Learning	Application	**-0.101 **(P < .001; N = 1591)	***0.203*** (P < .001; N = 1589)	**0.052 **(P = .038; N = 1584)
	Final Year	**-0.157 **(P < .001; N = 999)	**0.215** (P < .001; N = 1001)	**0.149 **(P < .001; N = 996)
SPQ Deep Learning	Application	**-0.078 **(P = .002; N = 1591)	**0.067 **(P = .007; N = 1589)	***0.192 ***(P < .001; N = 1584)
	Final Year	**-0.131 **(P < .001; N = 999)	**0.152 **(P < .001; N = 1001)	**0.240** (P < .001; N = 996)
General Health Questionnaire	PRHO	**0.169 **(P < .001; N = 974)	0.010 (P = .761; N = 974)	**-0.079 **(P = .014; N = 972)
	2002	**0.294** (P < .001; N = 1611)	0.018 (P = .471; N = 1608)	**-0.119 **(P < .001; N = 1604)
aMBQ – Emotional exhaustion	PRHO	0.062 (P = .103; N = 705)	-0.011 (P = .777; N = 704)	-0.042 (P = .267; N = 704)
	2002	**0.223** (P < .001; N = 1609)	0.007 (P = .782; N = 1607)	***-0.157 ***(P < .001; N = 1602)
aMBQ – Depersonalisation	PRHO	-0.003 (P = .939; N = 661)	-0.033 (P = .400; N = 658)	-0.017 (P = .672; N = 659)
	2002	**0.224** (P < .001; N = 1598)	**-0.071 **(P = .005; N = 1596)	**-0.151 **(P < .001; N = 1590)
aMBQ – Personal accomplishment	PRHO	**-0.137 **(P < .001; N = 938)	-0.032 (P = .335; N = 937)	**0.156 **(P < .001; N = 936)
	2002	0.024 (P = .335; N = 1604)	-0.013 (P = .610; N = 1602)	**0.095 **(P < .001; N = 1596)
Satisfaction with medicine as a career	2002	**-0.162 **(P < .001; N = 1620)	0.037 (P = .135; N = 1617)	**0.211** (P < .001; N = 1612)

**Table 5:** Pearson correlation (Significance; N) of background measures with the Workplace Climate Questionnaire. Correlations significant at p < 0.05 are in bold, and those with an absolute value of greater than 0.2 are underlined. Variables that are significant in the multiple regression (see text) are in italics.

	Time of measurement	aWCQ Workload	aWCQ Supportive-Receptive	aWCQ Choice-Independence
SPQ Surface Learning	Application	0.022 (P = .389; N = 1587)	0.042 (P = .093; N = 1578)	0.004 (P = .874; N = 1591
	Final Year	0.037 (P = .240; N = 999)	-0.034 (P = .287; N = 998)	0.054 (P = .090; N = 1000)
SPQ Strategic Learning	Application	0.032 (P = .196; N = 1591)	0.043 (P = .090; N = 1578)	**0.082 **(P = .001; N = 1591)
	Final Year	**0.063 **(P = .048; N = 999)	0.020 (P = .520; N = 998)	0.036 (P = .255; N = 1000)
SPQ Deep Learning	Application	0.008 (P = .738; N = 1587)	0.029 (P = .251; N = 1578)	0.049 (P = .051; N = 1591)
	Final Year	-0.032 (P = .306; N = 999)	**0.071 **(P = .026; N = 998)	**0.065 **(P = .041; N = 1000)
General Health Questionnaire	PRHO	**0.183 **(P < .001; N = 973)	**-0.134 **(P < .001; N = 970)	**-0.110 **(P = .001; N = 972)
	2002	***0.354*** (P < .001; N = 1606)	***-0.239*** (P < .001; N = 1597)	***-0.259*** (P < .001; N = 1609)
aMBQ – Emotional exhaustion	PRHO	**0.211** (P < .001; N = 703)	-0.056 (P = .136; N = 701)	-0.035 (P = .348; N = 703)
	2002	***0.400*** (P < .001; N = 1607)	**-0.164 **(P < .001; N = 1607)	**-0.220** (P < .001; N = 1609)
aMBQ – Depersonalisation	PRHO	0.057 (P = .145; N = 658)	**-0.109 **(P = .005; N = 656)	-0.020 (P = .609; N = 658)
	2002	**0.216** (P < .001; N = 1595)	***-0.222*** (P < .001; N = 1594)	**-0.165 **(P < .001; N = 1597)
aMBQ – Personal accomplishment	PRHO	-0.028 (P = .393; N = 937)	**0.110 **(P < .001; N = 933)	0.063 (P = .055; N = 936)
	2002	**0.163 **(P < .001; N = 1601)	***0.143 ***(P < .001; N = 1600)	0.044 (P = .079; N = 1603)
Satisfaction with medicine as a career	2002	**-0.256** (P < .001; N = 1617)	**0.317** (P < .001; N = 1616)	**0.294** (P < .001; N = 1620)

**Table 6:** Pearson correlation (Significance; N) of Approach to Work Questionnaire and the Workplace Climate Questionnaire with personality. Correlations significant at p < 0.05 are in bold, and those with an absolute value of greater than 0.2 are underlined. Variables that are significant in the multiple regression (see text) are in shaded boxes.

	Time of measurement	aAWQ Surface-Disorganised	aAWQ Surface-Rational	aAWQ Deep	aWCQ Workload	aWCQ Supportive-Receptive	aWCQ Choice-Independence
Neuroticism	PRHO	**0.172 **(P < .001; N = 993)	0.021 (P = .510; N = 993)	-0.050 (P = .113; N = 992)	**0.111 **(P < .001; N = 992)	-0.055 (P = .086; N = 989)	-0.017 (P = .592; N = 991)
	2002	***0.335*** (P < .001; N = 1645)	0.021 (P = .405; N = 1643)	**-0.111 **(P < .001; N = 1638)	**0.307** (P < .001; N = 1642)	**-0.180 **(P < .001; N = 1632)	**-0.168 **(P < .001; N = 1644)
Extraversion	PRHO	**-0.113 **(P < .001; N = 991)	0.056 (P = .079; N = 991)	0**.174 **(P < .001; N = 989)	**-0.090 **(P = .004; N = 990)	**-0.087 **(P = .006; N = 987)	0.049 (P = .124; N = 989)
	2002	**0.173 **(P < .001; N = 1649)	0.037 (P = .133; N = 1647)	***0.288*** (P < .001; N = 1643)	**-0.164 **(P < .001; N = 1647)	**0.147 **(P < .001; N = 1637)	**0.119 **(P < .001; N = 1649)
Openness to experience	PRHO	-0.011 (P = .729; N = 975)	**-0.077 **(P = .016; N = 975)	**0.263** (P < .001; N = 973)	-0.037 (P = .250; N = 974)	0.036 (P = .259; N = 971)	0.033 (P = .297; N = 973)
	2002	**-0.063 **(P = .010; N = 1646)	***-0.108 ***(P < .001; N = 1644)	***0.346*** (P < .001; N = 1639)	***-0.096 ***(P < .001; N = 1643)	0.038 (P = .129; N = 1633)	**0.080 **(P < .001; N = 1645)
Agreeableness	PRHO	-0.034 (P = .285; N = 991)	0.048 (P = .130; N = 991)	0.038 (P = .235; N = 989)	-0.024 (P = .455; N = 990)	***0.182 ***(P < .001; N = 987)	0.026 (P = .421; N = 989)
	2002	**-0.108 **(P < .001; N = 1650)	0.096 (P < .001; N = 1648)	0.038 (P = .121; N = 1643)	**-0.057 **(P = .021; N = 1647)	**0.153 **(P < .001; N = 1638)	0.017 (P = .498; N = 1650)
Conscientiousness	PRHO	***-0.353*** (P < .001; N = 991)	0.124 (P < .001; N = 991)	**0.084 **(P = .008; N = 989)	0.023 (P = .467; N = 991)	0.057 (P = .074; N = 987)	0.056 (P = 077; N = 990)
	2002	***-0.477*** (P < .001; N = 1645)	*0.126 *(P < .001; N = 1643)	**0.136 **(P < .001; N = 1638)	**-0.088 **(P < .001; N = 1641)	**0.093 **(P < .001; N = 1632)	**0.092 **(P < .001; N = 1644)

**Table 7:** Pearson correlation (Significance; N) of stress, burnout and satisfaction with medicine as a career in 2002 with study habits at application to medical school and in th final year of medical school, and with personality in the PRHO year and in 2002. Correlations significant at p < 0.05 are in bold, and those with an absolute value of greater than 0.2 are underlined. Variables that are significant in the multiple regression (see text) are in shown in italics.

	Time of measurement	Stress (GHQ) (2002)	Emotional exhaustion (aMBI) (2002)	Depersonalisation (aMBI)	Personal accomplishment (aMBI)	Satisfaction with medicine as a career
SPQ Surface Learning	Application	0.036 (P = .150; N = 1555)	**0.105 **(P < .001;N = 1552)	**0.068 **(P = .008;N = 1542)	-0.003 (P = .895;N = 1546)	-0.033 (P = .186;N = 1563)
	Final Year	0.008 (P = .792;N = 984)	**0.090 **(P = .005;N = 983)	**0.094 **(P = .003;N = 976)	-0.036 (P = .266;N = 980)	-0.023 (P = .471;N = 989)
SPQ Strategic Learning	Application	-0.011 (P = .671; N = 1555)	0.017 (P = .513;N = 1552)	**-0.073 **(P = .004;N = 1542)	0.021 (P = .416;N = 1546)	0.046 (P = .068;N = 1563)
	Final Year	-0.014 (P = .655;N = 984)	-0.052 (P = .102;N = 983)	**-0.108 **(P = .001;N = 976)	0.007 (P = .819;N = 980)	**0.104 **(P = .001;N = 989)
SPQ Deep Learning	Application	0.041 (P = .102; N = 1555)	-0.001 (P = .974;N = 1552)	**-0.055 **(P = .031;N = 1542)	***0.123 ***(P < .001;N = 1546)	0.042 (P = .101;N = 1563)
	Final Year	-0.022 (P = .498;N = 984)	**-0.105 **(P = .001;N = 983)	**-0.071 **(P = .027;N = 976)	**0.120 **(P < .001;N = 980)	**0.134 **(P < .001;N = 989)
Neuroticism	PRHO	**0.192 **(P < .001;N = 972)	**0.233 **(P < .001;N = 972)	**0.103 **(P = .001;N = 972)	0.036 (P = .255;N = 975)	**-0.188 **(P < .001;N = 981)
	2002	***0.461*** (P < .001; N = 1610)	***0.378*** (P < .001;N = 1607)	**0.235** (P < .001;N = 1596)	**0.090 **(P < .001;N = 1603)	***-0.314*** (P < .001;N = 1618)
Extraversion	PRHO	**-0.111 **(P = .001;N = 970)	**-0.190 **(P < .001;N = 970)	**-0.137 **(P < .001;N = 969)	**0.077 **(P = .016;N = 972)	**0.228 **(P < .001;N = 979)
	2002	**-0.243** (P < .001; N = 1614)	***-0.262*** (P < .001;N = 1611)	**-0.171 **(P < .001;N = 1599)	***0.152 ***(P < .001;N = 1606)	**0.307** (P < .001;N = 1621)
Openness to experience	PRHO	0.012 (P = .721;N = 956)	0.017 (P = .604;N = 955)	0.009 (P = .777;N = 954)	**0.096 **(P = .003;N = 957)	0.042 (P = .194;N = 964)
	2002	-0.046 (P = .066; N = 1611)	0.010 (P = .677;N = 1607)	-0.028 (P = .262;N = 1596)	**0.127 **(P < .001;N = 1603)	**0.066 **(P = .008;N = 1618)
Agreeableness	PRHO	-0.028 (P = .376;N = 970)	-0.062 (P = .055;N = 970)	**-0.240 **(P < .001;N = 969)	**0.091 **(P = .005;N = 972)	**0.101 **(P = .002;N = 979)
	2002	**-0.080 **(P = .001; N = 1615)	**-0.094 **(P < .001;N = 1611)	***-0.322*** (P < .001;N = 1600)	**0.082 **(P = .001;N = 1607)	**0.135 **(P < .001;N = 1622)
Conscientiousness	PRHO	-0.045 (P = .165;N = 971)	**-0.088 **(P = .006;N = 970)	**-0.121 **(P < .001;N = 969)	0.010 (P = .767;N = 972)	0.045 (P = .160;N = 979)
	2002	***-0.196 ***(P < .001; N = 1610)	**-0.129 **(P < .001;N = 1606)	**-0.165 **(P < .001;N = 1595)	0.031 (P = .209;N = 1602)	**0.162 **(P < .001;N = 1617)
