# Supplementary material for: Stress, burnout and doctors' attitudes to work are determined by personality and learning style: A twelve year longitudinal study of UK medical graduates
Source: BMC Med. 2004 Aug 18;2:29. doi: 10.1186/1741-7015-2-29 (PMC516448; doi:10.1186/1741-7015-2-29)
Supplement: Additional file 1 [file 1741-7015-2-29-S1.pdf]

Supplementary material

The abbreviated Approaches to Work Questionnaire (aAWQ).

The Approaches to Work Questionnaire described by Delva *et al* [1] had a total of thirty questions, ten on each of the three sub-scales. Based on their factor analysis [2] we reduced the scale to twelve items, four on each of the three scales. Table 1 shows the questions in the order in which they were presented. Factor analysis was carried out (principle component analysis followed by Varimax rotation). Three factors were extracted, that number being supported by a scree-slope analysis, the eigen-values being 2.068, 1.739, 1.532, 1.021, .891, .810, .787, .717, .668, .609, .596, and .562. The table shows the loadings on the three factors; absolute loadings less than 0.2 have been omitted for clarity. The pattern of loadings corresponds well with those described by Delva *et al* [1,2].

Table 1: Loadings of the twelve items of the aAWQ on the three factors.

	Factor 1: "Deep"	Factor 2: "Surface disorganised"	Factor 3: "Surface rational"
i. At work I find it difficult to organise my time effectively		.715	
ii. I find that studying new things can often be really exciting	.640	-.216	
iii. When I have something to do at work, I like to know precisely what is expected			.679
iv. Some of the issues that crop up at work are so interesting that I pursue them though they are not part of my job	.751		
v. My habit of putting off work leaves me with far too much catching up to do		.718	
vi. I prefer the work I am doing to be clearly structured			.688
vii. I like to play around with ideas of my own even if they don't get me very far	.608		-.229
viii. Although I generally remember facts and details, I find it difficult to fit them together into an overall picture		.613	
ix. I think it's important to look at problems rationally and logically without making intuitive jumps			.538
x. I spend a good deal of my spare time learning about things related to my work	.646		
xi. Often I have to read things without having a chance to really understand them		.544	.231
xii. When I learn something new at work I put a lot of effort into memorising important facts	.293		.485

The rubric for the questions said, "How well do the following statements describe your work style?", and the answer columns were headed Definitely disagree, Somewhat disagree, Somewhat agree, and Definitely agree, which were scored 1, 2,3 and 4 respectively. No items were reverse scored. Questions are in the order in which they were used in the questionnaire. Deep approach is scored as the sum of items 2, 4, 7 and 10. Surface-disorganised approach is the sum of items 1,5,8 and 11. Surface-rational approach is the sum of items 3,6,9 and 12. All scores must be in the range 4 to 16. For the 2002 cohort, the means (standard deviation; range) of the scores are: Deep: 11.14 (1.97; 5-16); Surface-disorganised: 8.09 (2.02; 4-14); Surface-rational 11.47 (1.72; 5-16). Distributions of scores are shown in figure 1.The alpha reliability coefficients of the scales are .584 (Deep), .469 (Surface-Rational), and .564 (Surface-Disorganised). For scales based on ten items, Delva *et al* reported values of .68, .74 and .63.

The abbreviated Workplace Climate Questionnaire (aWCQ).

The Workplace Climate Questionnaire described by Delva *et al* [1] had a total of thirty questions, ten on each of the three sub-scales. Based on their factor analysis [2] we reduced the scale to nine items, three on each of the three scales. Table 2 shows the questions in the order in which they were presented. Factor analysis was carried out (principle component analysis followed by Varimax rotation). Three factors were extracted, that number being supported by a scree-slope analysis, the eigen-values being 2.847, 1.636, 1.192, .757, .742, .693, .528, .356 and .248.The table shows the loadings on the three factors; absolute loadings less than 0.2 have been omitted for clarity. The pattern of loadings corresponds well with those described by Delva *et al* [1,2].

Table 2:

	Factor 1: "Choice-Independence"	Factor 2: "Workload"	Factor 3: "Supportive-Receptive"
xiii. There is a real opportunity in my job for me to choose the particular things I work on	.798		
xiv. My work colleagues really try hard to get to know one another			.778
xv. My work load is too heavy	-.237	.825	
xvi. I have a lot of choice about the work I do	.862		
xvii. The non-medical people I work with make a real effort to understand the difficulties doctors have with their work	.251		.600
xviii. My job requires me to do too many different things		.702	
xix. I pretty much decide how to do my work	.672		
xx. My coworkers are supportive and friendly towards me			.784
xxi. There seems to be too much work to get through in my job		.884	

The rubric for the questions said, "How well do the following statements describe your work style?", and the answer columns were headed Definitely disagree, Somewhat disagree, Somewhat agree, and Definitely agree, which were scored 1, 2,3 and 4 respetively. No items were reverse scored. Questions are in the order in which they were used in the questionnaire, where they followed the twelve questions of the aAWQ. Choice-independence is scored as the sum of items 13, 16 and 19. Workload is the sum of items 15, 18 and 21. Supportive-receptive is the sum of items 14, 17 and 20. All scores must be in the range 3 to 12. For the 2002 cohort, the means (standard deviation; range) of the scores are: Choice-Independence: 7.31 (1.96; 3-12); Workload: 7.34 (2.12; 3-12); Supportive-Receptive 8.38 (1.72; 3-12). Distributions of scores are shown in figure 1. The alpha reliability coefficients of the scales are .725 (Choice-Independence), ..744 (Workload), and .571 (Supportive-Receptive). For scales based on ten items, Delva *et al* reported values of .68, .70 and .56.

Distributions of scores on the aAWQ and aWCQ.

The histograms show the distributions of scores on the three sub-scales of the aAWQ and the aWCQ. The aAWQ has scores in the range 4 to 16, whereas the aWCQ has scores in the range 3 to 12.

Figure 1: Distributions of abbreviated Approach to Work Questionnaire and abbreviated Workplace Climate Questionnaire.

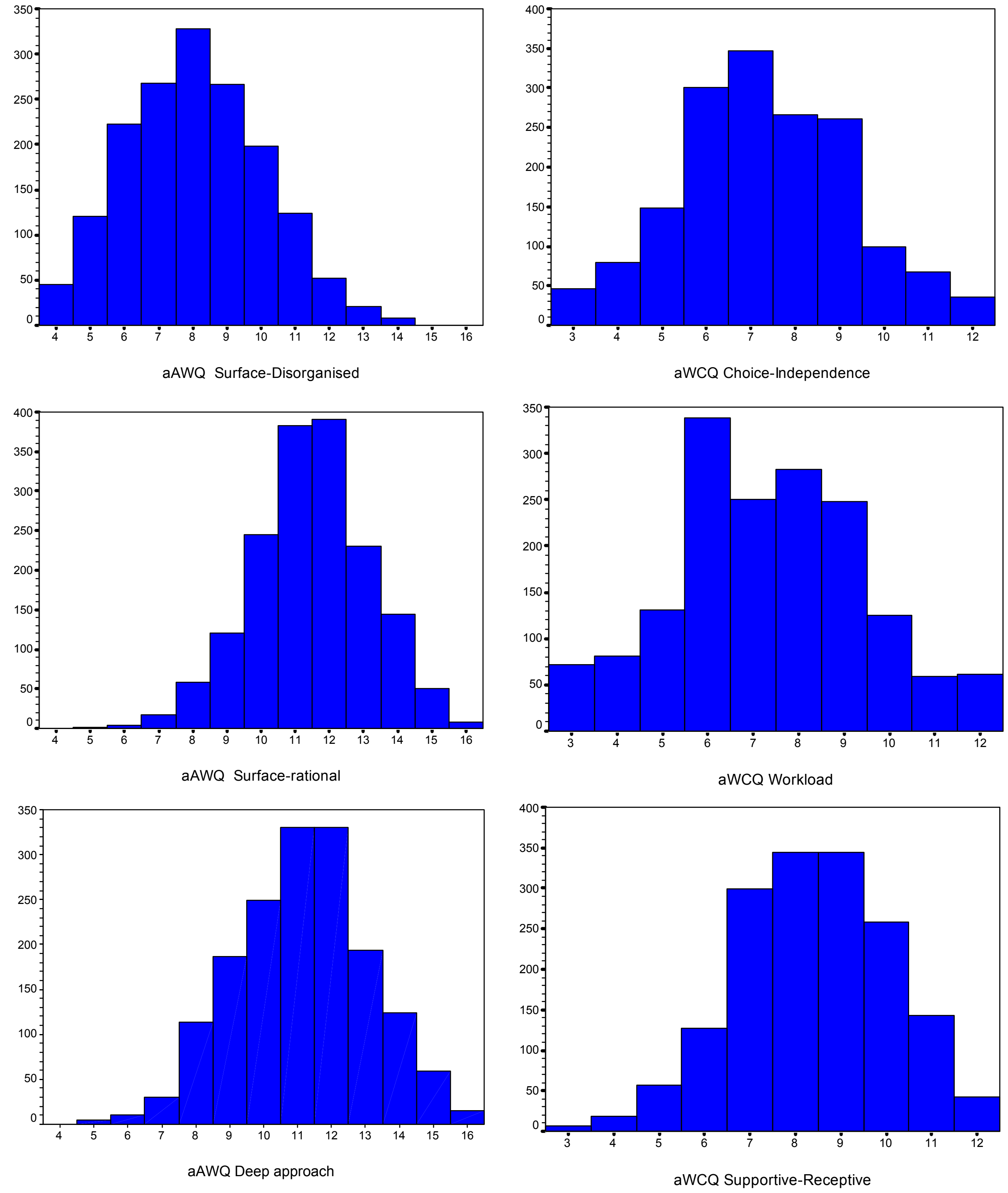

Response bias

The response rate in the present study was 63%, raising the possibility that those who responded to the survey were systematically different in important ways from those who did not respond. We investigated that possibility by comparing respondents and non-respondents in their responses to questions asked in previous surveys.

Of 2623 doctors sent questionnaires in the 2002 follow-up, 2501 (95.3%) had returned questionnaires in the survey of medical student selection in 1990, and of these 1615 (64.6%) returned the 2002 questionnaire, and 886 (35.4%) did not. Table 3 compares these groups on measures of study habits. Respondents had somewhat lower scores on all three measures, with the largest difference being on the measure of deep learning.

Table 3:

Measures at application in 1990 (mean (SD;N))	Respondents in 2002	Non-Respondents in 2002	Significance
Surface Learning	13.03 (3.62; 1605)	13.33 (3.91; 881)	t=1.96, 2484 df, p=0.050
Strategic Learning	22.71 (4.22; 1605)	23.14 (4.18; 881)	t=2.43, 2484 df, p=.015
Deep Learning	20.90 (4.15; 1605)	21.67 (4.39; 1605)	t=4.34, 2484 df, p<.001

Not all students had taken part in the final year follow-up study (in 1995 and 1996). Of the 1325 who returned the final year survey, 1019 (76.9%) had also returned the 2002 survey, and 306 (23.1%) had not. Table 4 compares these groups on measures of study habits. There were no significant differences between respondents and non-respondents.

Table 4:

Measures in final survey in 1995/6 (mean (SD;N))	Respondents in 2002	Non-Respondents in 2002	Significance
Surface Learning	14.00 (3.74; 1009)	14.13 (4.01; 304)	t=0.55, 1311 df, p=0.138
Strategic Learning	15.26 (5.01; 1009)	15.01 (5.05; 304)	t= –.766, 1311 df, p=.444
Deep Learning	18.34 (4.33; 1009)	18.68 (4.70; 304)	t=1.16, 1311 df, p=.245

1293 doctors who had replied to the questionnaire survey in 1996/1997 of PRHOs were sent the 2002 questionnaire, and of these 1007 (77.9%) replied and 286 (22.1%) did not. Table 5 compares the two groups in terms of stress, burnout and the five personality dimensions as measured at the time of the PRHO survey. None of the measures showed any significant differences.

Table 5:

Measures in PRHO survey in 1996/7 (mean (SD;N))	Respondents in 2002	Non-Respondents in 2002	Significance
GHQ score	12.49 (5.53; 982)	12.07 (5.31; 270)	t= –1. 13, 1250 df, p=.268
Emotional exhaustion (aMBI)	10.92 (2.93;982)	11.15 (2.91; 270)	t= 1.02, 1250 df, p=.308
Depersonalisation (aMBI)	10.29 (3.37;982)	10.65 (3.22; 270)	t= 1.26, 1250 df, p=.209
Personal accomplishment (aMBI)	13.07 (2.61; 946)	13.21 (2.65; 258)	t= .76, 1202 df p=.450
Neuroticism	8.87 (2.25; 1001)	8.88 (2.20; 280)	t= .082, 1279 df p=.935
Extraversion	10.57 (1.89; 999)	10.58 (1.85; 282)	t= .075, 1279 df p=.941
Openness to experience	12.23 (2.36; 983)	12.35 (2.42; 279)	t= .705, 1260 df p=.481
Agreeableness	14.67 (1.62; 999)	14.57 (1.63; 279)	t= –.954, 1276 df p=.340
Conscientiousness	13.74 (1.79; 999)	13.66 (1.80; 282)	t= –.708, 1279 df p=.479

Taken overall there is very little suggestion of response bias, with the exception that respondents in 2002 had slightly lower deep learning scores at application than did non-respondents. Such a difference is unlikely to have any impact on the conclusions reached in the main paper, not least because the analyses are correlational, rather than comparing means of groups.

Multiple regression analyses.

The various correlations in main text tables 4 to 6 are not always straightforward to interpret, not only because of their number, but because many of the variables which one wishes to treat as 'independent variables' are themselves correlated, as are the variables which one is interested in as dependent variables. Multiple regression was used to clarify the relationships. One at time, each of the three measures of the aAWQ and aWCQ was used as the dependent variable, and the other five measures in the set used as dependent variables were then entered into the equation, to remove any effects due the correlation between the measures themselves. Forward entry regression was then used to find those independent variables which predicted that dependent variable, from the measures of study habits at application (n=3) and in the final year (n=3), of stress and burnout during the PRHO year (n=4) and in 2002 (n=4), and of personality in the PRHO year (n=5) and in 2002 (5). In view of the large sample size and the number of variables, and the desire to find the most important relationships, the entry criterion was set at $p<.0001$. Variables which are significant using this method are shaded in main text tables 4, 5 and 6. Since the particular emphasis was on correlations across time, variables are only described in the main text if the contemporaneous correlation is significant, and also the time-lagged correlation between a measure in 2002 and a measure at a previous time point is significant.

The surface-disorganised approach to work was predicted by surface learning at application to medical school (β =.076, t=3.76), by lower conscientiousness (independent effects when measured in the PRHO year (β = –.140, t=–6.70) and in 2002 (β = –.392, t=18.17)), and by greater neuroticism (β =.205, t=9.51). The surface-rational approach was predicted by strategic learning at application (β =.161, t=6.59), by higher conscientiousness (β =.114, t=4.13), and by lower openness to experience (β = –.131, t= –5.14). A deep approach to work was predicted by deep learning at application to medical school (β =.117; t=5.32), by higher openness to experience (β =.301; t=13.26), by higher extraversion (β =.171, t=7.28), and by less emotional exhaustion (β =-.103, t= –4.18). Approaches to work are therefore predicted mainly by previous study habits and by personality measures, particularly conscientiousness and openness to experience. In contrast, workplace climate measures are predicted mainly by measures of stress and burnout. A high perceived workload is predicted by greater emotional exhaustion (β =.286, t=11.94) and by a high GHQ score (β = .173, t=7.03), and by lower openness to experience (β = –.091, t= –3.92). A more supportive-receptive environment is predicted by higher personal accomplishment (β =.157, t=6.87), by lower depersonalisation (β = –.127, t= –5.26), and by a lower GHQ score (β = -.119, t= –4.75), as well as by higher agreeableness (β = .099, t=4.36). Choice-independence is predicted only by a lower GHQ score (β = – .125, t= –5.04).

As well as being treated as predictor variables in the previous paragraph, the measures of stress and burnout were also treated as dependent variables in their own right. Each of the five 'stress' measures was used in turn as dependent variable, and the other four stress measures entered into the analysis to remove any effect due to correlations between the set of dependent variables. Forward entry regression was then used to find predictors of the dependent variable, from the background measures of study habits at application (3) and in the final year (3) and personality in the PRHO year (5) and in 2002 (5). The entry criterion was set at $p<.0001$.

Stress, as measured by the GHQ, was predicted by higher neuroticism (β =.321, t=13.92), and by lower conscientiousness (β = –.088, t= –4.12). Emotional exhaustion was predicted by higher neuroticism (β = .105, t= –4.96) and introversion (β = –.131, t= –6.69), and the inverse pattern was showed by satisfaction with medicine as a career, which was predicted by lower neuroticism (β = –.108, t= –4.49) and extraversion (β =.096, t=4.30). Depersonalisation was predicted by low scores on the personality trait of agreeableness (β = –.261, t= –12.48), and a higher sense of personal accomplishment was predicted by extraversion (β =.176, t=7.59) and a higher deep learning score (β =.093, t=4.26). In all but one case, when a variable was a significant

predictor there was also a highly significant prediction in the same measure on an earlier occasion.

It should be noticed that in all of the above analyses, the correlations reported are particularly ‘pure’ in the sense that although normally measures of stress, and the burnout sub-scales are inter-correlated, the analysis means that, say, any component of stress, emotional exhaustion, personal accomplishment and satisfaction with medicine as a career has been removed from the measure of depersonalisation. It is for that reason that depersonalisation only relates to agreeableness, the simple correlations with neuroticism, extraversion and conscientiousness being shared with other aspects of stress and burnout.

Structural Equation Modelling

Structural equation modelling (SEM) was carried out with the four stress variables (GHQ, and the three burnout measures) represented only by the single GHQ measure, since it correlates highly with the other three measures. That is the version of the model presented in the main paper. However data for the three burnout measures are also presented in the correlation matrix at the end of the Supplementary Information for readers who are interested in reworking a more complex model.

Causal ordering. The causal ordering of variables is path analysis is always potentially controversial. As is conventional in the SEM literature, the program and the correlation matrix are therefore presented below, so that those interested can rework the model using different assumptions. Causal ordering, the principles of which are described elsewhere [3-6], relies on a mixture of temporal ordering, logical necessity, and theoretical understanding, particularly when that understanding is based in previous empirical research. In the present case, there is general agreement in the literature that the majority of personality variance is stable across the life-span, and hence the five personality measures are placed prior to other variables. Learning style measures follow, both because they are temporally early, being assessed during medical school, and because they also have moderate long-term stability; they follow personality, because it seems more reasonable to propose that personality determines learning style rather than vice-versa. The measures of work environment are placed late in the causal hierarchy, both because they are temporally late, employment occurring after training, and because they are known to correlate with personality and learning style measures assessed far earlier in time, and hence are probably caused by those earlier variables. Approaches to Work are assumed to have more long-term stability than Workplace Climate, and hence Climate is placed after Approaches to work. Stress has been measured on two separate occasions in our study, and previous stress in the PRHO year correlates with subsequent measures of Workplace Climate and Workplace Learning suggesting that a stress response causes Workplace measures, rather than vice-versa. We have therefore placed Stress before the Workplace measures.

Variables. Although a number of variables have been measured on two separate occasions, including both measures would unduly complicate the presentation of the model (which is already complex enough as it is). We have therefore chosen to reduce our several measures of personality, learning style and stress/burnout, to a single measure. For most subjects we have averaged the two separate variables which assess each measure. If subjects provided only one measure, because of questionnaire non-response, then that measure was used as the estimate of personality, learning style or stress. Missing values otherwise were handled by mean substitution. The full correlation matrix is shown at the end of the supplementary information.

The LISREL program. The raw data are represented in LISREL as Y variables, the directed, causal effects are placed in the *beta* (**B**) matrix, and the undirected synchronous correlations between variables measured at the same time are placed in the *psi* (Ψ) matrix. The *lambda* (Λ) matrix is a

fixed, identity matrix, and the error matrix, *theta-epsilon* (θ_ϵ) is diagonal and free. The data analysed consist of the correlation matrix, and hence the diagonals of the *psi* matrix are fixed at one. Model fitting proceed by beginning with a fully saturated model in which all off-diagonal, synchronous correlations in the *psi* matrix were free, and the *beta* matrix was saturated in the sense that all variables to the left could have a causal influence on all other variables to the right. Non-significant *beta* paths were then removed from the model sequentially, removing the least significant first, and re-estimating the model. In the final model all *beta* paths included in the model were significant with $p<.05$ ($|t| >1.96$). That non-included *beta* paths were indeed non-significant was confirmed by individually re-entering into the model each of those with high modification indices. The program shown below is that which was used for the final fitted model as described in the main paper. Abbreviations of variable names are shown at the end of **the Supplementary Information**.

```
McManus et al, Stress Burnout and Approaches to Work - simplified model
DA NI=18 NO=1660 ma=km
[Insert instructions here to read the correlation matrix]
la
NEUR EXTRA OPEN AGREE CONSC SURF STRT DEEP STRESS EE DP PA AWQDeep AWQSR
AWQSD WCQCI WCQWL WCQSR
se
NEUR EXTRA OPEN AGREE CONSC SURF STRT DEEP STRESS AWQDeep AWQSR AWQSD
WCQCI WCQWL WCQSR /
MO NY=15 ps=sy,fr te=ze be=fu,fr
awdeep awsrat awsdis cind wload supprec  /
MO NY=15 ps=sy,fr te=ze be=fu,fr
pa ps
*
1
1 1
1 1 1
1 1 1 1
1 1 1 1 1
0 0 0 0 0  1
0 0 0 0 0  1 1
0 0 0 0 0  1 1 1
0 0 0 0 0  0 0 0  1
0 0 0 0 0  0 0 0  0  1
0 0 0 0 0  0 0 0  0  1 1
0 0 0 0 0  0 0 0  0  1 1 1
0 0 0 0 0  0 0 0  0  0 0 0  1
0 0 0 0 0  0 0 0  0  0 0 0  1 1
0 0 0 0 0  0 0 0  0  0 0 0  1 1 1
pa be
*
0 0 0 0 0  0 0 0  0  0 0 0  0 0 0
0 0 0 0 0  0 0 0  0  0 0 0  0 0 0
0 0 0 0 0  0 0 0  0  0 0 0  0 0 0
0 0 0 0 0  0 0 0  0  0 0 0  0 0 0
0 0 0 0 0  0 0 0  0  0 0 0  0 0 0
0 1 1 1 0  0 0 0  0  0 0 0  0 0 0
0 1 1 1 1  0 0 0  0  0 0 0  0 0 0
0 1 1 0 1  0 0 0  0  0 0 0  0 0 0
1 1 0 1 1  0 0 1  0  0 0 0  0 0 0
0 1 1 0 0  0 1 1  1  0 0 0  0 0 0
0 1 1 0 1  1 1 1  0  0 0 0  0 0 0
1 0 0 0 1  1 0 1  1  0 0 0  0 0 0
0 0 0 0 0  0 0 0  1  1 0 0  0 0 0
1 0 1 0 1  0 1 0  1  0 0 1  0 0 0
0 0 0 1 0  0 0 0  1  1 1 0  0 0 0
OU me=ml MI RS EF MR SS SC  nd=3
```

The table of *beta* coefficients below shows *all* paths which were significant with p<.05. However for simplicity, the path diagram shown in the main paper includes only those paths which have |t| > 3.6 (equivalent to p≅.0005). Those interested in other non-drawn paths may find them in the table below. For parameters included in the model, LISREL displays firstly the parameter estimate, then the standard error in parentheses, and finally the t-statistic (which for all intents and purposes is equivalent to a z statistic, given the large sample size).

PSI

	NEUR	EXTRA	OPEN	AGREE	CONSC	SURF
NEUR	1.000					
	(0.035)					
	28.801					
EXTRA	-0.267	1.000				
	(0.025)	(0.035)				
	-10.507	28.801				
OPEN	-0.029	0.214	1.000			
	(0.025)	(0.025)	(0.035)			
	-1.181	8.523	28.801			
AGREE	-0.101	0.123	0.096	1.000		
	(0.025)	(0.025)	(0.025)	(0.035)		
	-4.093	4.972	3.892	28.801		
CONSC	-0.120	0.177	0.020	0.193	1.000	
	(0.025)	(0.025)	(0.025)	(0.025)	(0.035)	
	-4.853	7.099	0.814	7.719	28.801	
SURF	- -	- -	- -	- -	- -	0.973
						(0.034)
						28.801
STRT	- -	- -	- -	- -	- -	0.085
						(0.023)
						3.651
DEEP	- -	- -	- -	- -	- -	-0.181
						(0.024)
						-7.513
STRESS	- -	- -	- -	- -	- -	- -
AWQDeep	- -	- -	- -	- -	- -	- -
AWQSR	- -	- -	- -	- -	- -	- -
AWQSD	- -	- -	- -	- -	- -	- -
WCQCI	- -	- -	- -	- -	- -	- -
WCQWL	- -	- -	- -	- -	- -	- -
WCQSR	- -	- -	- -	- -	- -	- -

PSI

	STRT	DEEP	STRESS	AWQDeep	AWQSR	AWQSD
STRT	0.927					
	(0.032)					
	28.801					
DEEP	0.398	0.955				
	(0.025)	(0.033)				
	15.885	28.801				
	STRT	DEEP	STRESS	AWQDeep	AWQSR	AWQSD
STRESS	- -	- -	0.730			
			(0.025)			
			28.801			
AWQDeep	- -	- -	- -	0.818		
				(0.028)		
				28.801		
AWQSR	- -	- -	- -	0.099	0.928	
				(0.022)	(0.032)	
				4.587	28.801	
AWQSD	- -	- -	- -	0.019	0.054	0.718
				(0.019)	(0.020)	(0.025)
				0.983	2.684	28.801
WCQCI	- -	- -	- -	- -	- -	- -
WCQWL	- -	- -	- -	- -	- -	- -
WCQSR	- -	- -	- -	- -	- -	- -

```
  PSI
         WCQCI      WCQWL      WCQSR
       --------   --------   --------
WCQCI      0.913
         (0.032)
          28.801

WCQWL     -0.139      0.844
         (0.022)    (0.029)
          -6.376     28.801

WCQSR      0.280     -0.082      0.920
         (0.024)    (0.022)    (0.032)
          11.919     -3.795     28.801
```

LISREL provides a wide range of Goodness of Fit statistics for models, which are shown below. Although the chi-square statistic itself just reaches significance (64.85, 43 df, p=.0172), that is hardly surprising given the large sample size. Probably the most useful measures of the success of the model are that the Goodness of Fit Index (GFI) = 0.995 and the Adjusted Goodness of Fit Index (AGFI) = 0.986. Other measures of fit are shown below:

```
                         Degrees of Freedom = 43
            Minimum Fit Function Chi-Square = 64.855 (P = 0.0172)
    Normal Theory Weighted Least Squares Chi-Square = 64.450 (P = 0.0187)
          Estimated Non-centrality Parameter (NCP) = 21.450
      90 Percent Confidence Interval for NCP = (3.780 ; 47.082)

                    Minimum Fit Function Value = 0.0391
            Population Discrepancy Function Value (F0) = 0.0129
          90 Percent Confidence Interval for F0 = (0.00228 ; 0.0284)
           Root Mean Square Error of Approximation (RMSEA) = 0.0173
        90 Percent Confidence Interval for RMSEA = (0.00728 ; 0.0257)
             P-Value for Test of Close Fit (RMSEA < 0.05) = 1.00

                 Expected Cross-Validation Index (ECVI) = 0.132
          90 Percent Confidence Interval for ECVI = (0.121 ; 0.147)
                       ECVI for Saturated Model = 0.145
                     ECVI for Independence Model = 2.654
  Chi-Square for Independence Model with 105 Degrees of Freedom = 4373.235
                        Independence AIC = 4403.235
                            Model AIC = 218.450
                          Saturated AIC = 240.000
                       Independence CAIC = 4499.454
                           Model CAIC = 712.372
                         Saturated CAIC = 1009.749
                        Normed Fit Index (NFI) = 0.985
                     Non-Normed Fit Index (NNFI) = 0.987
                  Parsimony Normed Fit Index (PNFI) = 0.403
                     Comparative Fit Index (CFI) = 0.995
                     Incremental Fit Index (IFI) = 0.995
                       Relative Fit Index (RFI) = 0.964

                          Critical N (CN) = 1726.640
                  Root Mean Square Residual (RMR) = 0.0169
                         Standardized RMR = 0.0169
                     Goodness of Fit Index (GFI) = 0.995
                Adjusted Goodness of Fit Index (AGFI) = 0.986
               Parsimony Goodness of Fit Index (PGFI) = 0.356
```

R^2 values. The R^2 values for the variables in the structural model are shown below, and correspond to the proportion of the variance in each measure which is explained by the model.

```
     Squared Multiple Correlations for Structural Equations
           NEUR      EXTRA       OPEN      AGREE      CONSC       SURF
       --------   --------   --------   --------   --------   --------
           - -        - -        - -        - -        - -       0.027

           STRT       DEEP     STRESS    AWQDeep      AWQSR      AWQSD
       --------   --------   --------   --------   --------   --------
          0.075      0.044      0.269      0.184      0.073      0.280

          WCQCI      WCQWL      WCQSR
       --------   --------   --------
          0.088      0.154      0.081
```

Correlation matrix. The complete correlation matrix used for the LISREL analyses is shown below. It includes the three measures of burnout which were excluded from the simplified model presented in the main paper. Abbreviations are shown at the end of the Supplementary Information.

	NEUR	EXTRA	OPEN	AGREE	CONSC	SURF	STRT	DEEP	STRESS	EE	DP	PA	AWQDeep	AWQSR	AWQSD	WCQCI	WCQWL	WCQSR
NEUR	1.000	-0.267	-0.029	-0.101	-0.120	0.048	-0.013	-0.001	0.489	0.404	0.230	0.038	-0.110	0.017	0.314	-0.135	0.271	-0.150
EXTRA	-0.267	1.000	0.214	0.123	0.177	-0.120	0.068	0.161	-0.268	-0.278	-0.216	0.212	0.269	0.052	-0.167	0.099	-0.157	0.137
OPEN	-0.029	0.214	1.000	0.096	0.020	-0.121	-0.127	0.147	-0.009	0.040	0.035	0.171	0.325	-0.115	-0.052	0.065	-0.069	0.031
AGREE	-0.101	0.123	0.096	1.000	0.193	-0.075	-0.005	0.049	-0.028	-0.082	-0.284	0.148	0.022	0.068	-0.078	0.017	-0.034	0.159
CONSC	-0.120	0.177	0.020	0.193	1.000	-0.062	0.226	0.103	-0.158	-0.113	-0.130	0.111	0.104	0.106	-0.434	0.087	-0.055	0.078
SURF	0.048	-0.120	-0.121	-0.075	-0.062	1.000	0.084	-0.215	0.019	0.097	0.096	-0.011	-0.102	0.117	0.131	0.017	0.031	0.033
STRT	-0.013	0.068	-0.127	-0.005	0.226	0.084	1.000	0.409	-0.042	-0.035	-0.108	-0.006	0.099	0.209	-0.124	0.069	0.031	0.018
DEEP	-0.001	0.161	0.147	0.049	0.103	-0.215	0.409	1.000	0.015	-0.035	-0.103	0.133	0.232	0.101	-0.103	0.056	-0.007	0.043
STRESS	0.489	-0.268	-0.009	-0.028	-0.158	0.019	-0.042	0.015	1.000	0.445	0.273	0.006	-0.126	0.006	0.292	-0.235	0.331	-0.223
EE	0.404	-0.278	0.040	-0.082	-0.113	0.097	-0.035	-0.035	0.445	1.000	0.452	0.235	-0.134	-0.004	0.206	-0.190	0.378	-0.147
DP	0.230	-0.216	0.035	-0.284	-0.130	0.096	-0.108	-0.103	0.273	0.452	1.000	0.061	-0.140	-0.066	0.199	-0.119	0.179	-0.209
PA	0.038	0.212	0.171	0.148	0.111	-0.011	-0.006	0.133	0.006	0.235	0.061	1.000	0.118	-0.011	-0.012	0.054	0.130	0.151
AWQDeep	-0.110	0.269	0.325	0.022	0.104	-0.102	0.099	0.232	-0.126	-0.134	-0.140	0.118	1.000	0.101	-0.085	0.207	-0.067	0.101
AWQSR	0.017	0.052	-0.115	0.068	0.106	0.117	0.209	0.101	0.006	-0.004	-0.066	-0.011	0.101	1.000	0.023	-0.001	0.045	0.063
AWQSD	0.314	-0.167	-0.052	-0.078	-0.434	0.131	-0.124	-0.103	0.292	0.206	0.199	-0.012	-0.085	0.023	1.000	-0.125	0.258	-0.111
WCQCI	-0.135	0.099	0.065	0.017	0.087	0.017	0.069	0.056	-0.235	-0.190	-0.119	0.054	0.207	-0.001	-0.125	1.000	-0.227	0.346
WCQWL	0.271	-0.157	-0.069	-0.034	-0.055	0.031	0.031	-0.007	0.331	0.378	0.179	0.130	-0.067	0.045	0.258	-0.227	1.000	-0.168
WCQSR	-0.150	0.137	0.031	0.159	0.078	0.033	0.018	0.043	-0.223	-0.147	-0.209	0.151	0.101	0.063	-0.111	0.346	-0.168	1.000

Stress, burnout and satisfaction with medicine

We have chosen in the structural equation modelling to look only at a single outcome measure, 'stress', represented by the score on the GHQ-12, scored on a 0-1-2-3 basis. We did however also have measures of the three sub-scales (EE: Emotional exhaustion; DP: depersonalisation; PA: Personal accomplishment) of the abbreviated Maslach Burnout Inventory (aMBI). In the 2002 follow-up we also had a measure of 'satisfaction with medicine',which had three separate items [7]. The GHQ-12 and aMBI were administered to the doctors both as PRHOs (PRHO) and in 2002, while the Satisfaction measure (SAT) was only administered in 2002. Here we assess the inter-relationships between the measures and demonstrate that GHQ-12 provides a good surrogate for the other measures.

There was complete data on all nine measures for 464 respondents. The correlation matrix is shown below. All of the measures load positively with the GHQ measures (except for PA and SAT, which are scored in the reverse direction, so that high stress correlates with low personal accomplishment and satisfaction).

	GHQPRHO	GHQ2002	EEPRHO	EE2002	DPPRHO	DP2002	PAPRHO	PA2002	SAT2002
GHQPRHO	1.000	.356	.500	.280	.273	.185	-.255	-.125	-.268
GHQ2002	.356	1.000	.224	.504	.093	.315	-.048	-.173	-.420
EEPRHO	.500	.224	1.000	.376	.482	.172	-.004	-.026	-.258
EE2002	.280	.504	.376	1.000	.222	.542	-.059	-.033	-.506
DPPRHO	.273	.093	.482	.222	1.000	.288	-.017	-.105	-.201
DP2002	.185	.315	.172	.542	.288	1.000	-.041	-.060	-.365
PAPRHO	-.255	-.048	-.004	-.059	-.017	-.041	1.000	.345	.180
PA2002	-.125	-.173	-.026	-.033	-.105	-.060	.345	1.000	.312
SAT2002	-.268	-.420	-.258	-.506	-.201	-.365	.180	.312	1.000

Principle Component Analysis showed that the first component accounted for 34.1% of the total variance. The scree-slope analysis (below) showed a clear 'dog-leg' after the first factor, suggesting that all of the measures were loading on a single common factor, and indeed all the loadings were in the expected direction.

	Component
	1
EE2002	.758
SAT2002	-.703
GHQ2002	.652
GHQPRHO	.628
EEPRHO	.623
DP2002	.615
DPPRHO	.510
PA2002	-.303
PAPRHO	-.244

Scree Plot

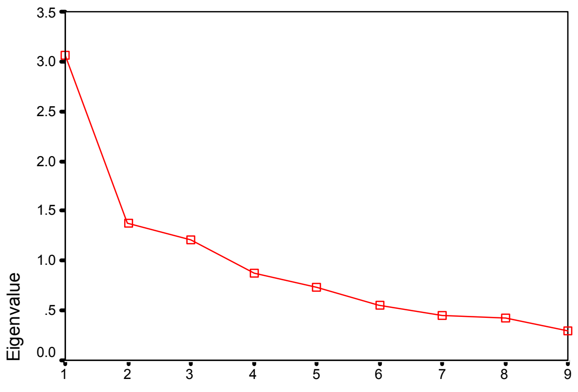

Although the correlation matrix does reveal some subtleties about the relationship between the nine measures, it is clear that the GHQ measure is a good proxy for the majority of the common variance within the measures.

Abbreviations

NEUR	Neuroticism (Big Five personality measure)
EXTRA	Extraversion (Big Five personality measure)
OPEN	Openness to experience (Big Five personality measure)
AGREE	Agreeableness (Big Five personality measure)
CONSC	Conscientiousness (Big Five personality measure)
SURF	Surface learning (Study Process Questionnaire)
STRT	Strategic learning (Study Process Questionnaire)
DEEP	Deep learning (Study Process Questionnaire)
STRESS	Stress (General Health Questionnaire, 0-1-2-3 scoring)
EE	Emotional Exhaustion (Maslach Burnout Inventory)
DP	Depersonalisation (Maslach Burnout Inventory)
PA	Personal Accomplishment (Maslach Burnout Inventory)
AWQDeep	Deep approach to work (Approaches to Work Questionnaire)
AWQSR	Surface-Rational approach to work (Approaches to Work Questionnaire)
AWQSD	Surface-Disorganised approach to work (Approaches to Work Questionnaire)
WCQCI	Choice-independence in work environment (Workplace Climate Questionnaire)
WCQWL	High workload (Workplace Climate Questionnaire)
WCQCI	Supportive-Receptive work environment (Workplace Climate Questionnaire)

Reference List

1. Delva MD, Kirby JR, Knapper CK, Birtwhistle RV: **Postal survey of approaches to learning among Ontario physicians: implications for continuing medical education.** *Brit Med J* 2002, **325:** 1218.

2. Kirby JR, Delva MD, Knapper CK, Birtwhistle RV: **Development of the Approaches to Work and Workplace Climate Questionnaires for Physicians.** *Evaluation and the Health Professions* 2003, **26:** 104-121.

3. Kenny DA: *Correlation and causality*. New York: John Wiley; 1979.

4. Davis JA: *The logic of causal order*. London: Sage; 1985.

5. Glymour C, Scheines R, Spirtes P, Kelly K: *Discovering causal structure*. Academic Press; 1987.

6. Pearl J: *Causality: Models, reasoning, and inference*. Cambridge: Cambridge University Press; 2000.

7. McManus IC, Smithers E, Partridge P, Keeling A, Fleming PR: **A levels and intelligence as predictors of medical careers in UK doctors: 20 year prospective study.** *Brit Med J* 2003, **327:** 139-142.
